# 

**Published:** 1893-11

**Authors:** 


CONTENTS.

Vol. XL. No. 11.

| | PAGE. |
|---|---|
| Avoidable Diseases. C. B. Newton, M. D. | 225 |
| The Nervous System. L. J. White. | 228 |
| Health and Civilization. W. W. Hall, M. D. | 232 |
| Miscellaneous Paragraphs | 235 |
| House and Home. Conducted by Lillian White | 238 |
| Editorial—Our Aim | 243 |
| Literary | 243 |
| General Notes | 250 |

